# Identification and Validation of QTLs for Macronutrient Contents in Brown and Milled Rice Using Two Backcross Populations between *Oryza sativa* and *O. rufipogon*

**DOI:** 10.1155/2021/5561734

**Published:** 2021-06-11

**Authors:** Biao-lin Hu, Xia Li, Ting Wu, De-run Huang, Feng-lin Huang, Jian-hua Yin, Yan-shou Wu

**Affiliations:** ^1^Rice Research Institute, Jiangxi Academy of Agricultural Sciences/National Engineering Laboratory for Rice (Nanchang), Nanchang 330200, China; ^2^State Key Laboratory of Rice Biology/Chinese National Center for Rice Improvement, China National Rice Research Institute, Hangzhou 310006, China; ^3^Ministry of Agriculture, Key Laboratory of Indica Rice Genetics and Breeding in the Middle and Lower Reaches of Yangtze River Valley, Changsha 410125, China

## Abstract

Mineral malnutrition as a prevalent public health issue can be alleviated by increasing the intake of dietary minerals from major staple crops, such as rice. Identification of the gene responsible for mineral contents in rice would help breed cultivars enriched with minerals through marker-assisted selection. Two segregating populations of backcross inbred lines (BIL) were employed to map quantitative trait loci (QTLs) for macronutrient contents in brown and milled rice, BC_1_F_5_, and BC_2_F_4:5_ derived from an interspecific cross of Xieqingzao B (*Oryza sativa*) and Dongxiang wild rice (*O*. *rufipogon*). Phenotyping the populations was conducted in multiple locations and years, and up to 169 DNA markers were used for the genotyping. A total of 17 QTLs for P, K, Na, Ca, and Mg contents in brown and milled rice distributed on eight regions were identified in the BC_1_F_5_ population, which is explained to range from 5.98% to 56.80% of phenotypic variances. Two regions controlling *qCa1.1* and *qCa4.1* were validated, and seven new QTLs for Ca and Mg contents were identified in the BC_2_F_4:5_ population. 18 of 24 QTLs were clustered across seven chromosomal regions, indicating that different mineral accumulation might be involved in common regulatory pathways. Of 24 QTLs identified in two populations, 16 having favorable alleles were derived from *O*. *rufipogon* and 10 were novel. These results will not only help understand the molecular mechanism of macronutrient accumulation in rice but also provide candidate QTLs for further gene cloning and grain nutrient improvement through QTL pyramiding.

## 1. Introduction

Mineral nutrients are vital for human health, and more than 25 minerals are required by humans. These minerals can be obtained directly from the daily diet in an appropriate amount [1], and the important minerals are phosphorus (P), sodium (Na), potassium (K), magnesium (Mg), calcium (Ca), iron (Fe), zinc (Zn), copper (Cu), and manganese (Mn). Macroelements, such as P, K, Na, Mg, and Ca, play a pivotal role in the metabolic functions of fluid balance, neurotransmission, blood pressure regulation, and human's immune system [2, 3]. Although the micronutrient Fe, Zn, and I deficiencies are very common worldwide, macronutrient Mg and Ca deficiencies are serious in some countries [4, 5] and spread across more countries recently [6, 7]. The deficiencies might be partly due to high dietary dependence on a few major staple cereals lacking adequate essential minerals.

Rice (*Oryza sativa* L.) is one of the most important staple foods for over half of the global population. Rice grains store macronutrients (P, K, Mg, and Ca) as well as micronutrients (Fe, Zn, Cu, and Mn) for seed germination and seedling growth [8–10]. Rice grain could be a source of energy and mineral nutrients for rice seedling and human development. Mineral deficiencies may be improved by enhancing grain mineral nutrients. However, most of modern high-yielding rice cultivars do not have sufficient essential minerals to meet the daily dietary requirement, especially in most preferred consumption of polished rice [11]. It is a growing demand to enhance essential mineral contents in rice grain through breeding-based biofortification approaches. Wide variations have been observed for grain mineral contents in diverse rice genotypes [12], which provide a desirable potential to breed rice varieties enriched with essential nutrient. Understanding the genetic basis of mineral accumulation in rice is imperative and will greatly facilitate the enhancement of mineral nutrient in the rice breeding program [13].

Quantitative trait locus (QTL) mapping is a powerful and desirable approach to explore the chromosomal regions controlling quantitative traits for the marker-assisted selection (MAS) strategy in rice. Up to now, a larger number of QTLs associated with mineral contents in rice have been identified using various mapping populations derived from biparental inter- or intra-subspecific and interspecific crosses [5, 11, 13–16]. Using the RILs from the intra-subspecific cross Zhenshan 97/Miyang 46, Yu et al. [13] identified 20 QTLs for P, K, Mg, Ca, Zn, Mn, and Cu contents in milled rice and Wang et al. [5] identified 51 and 61 QTLs for the contents of 13 and 15 minerals including P, K, Na, Mg, and Ca in brown rice and straw, respectively. Garcia-Oliveira et al. [15] identified 31 QTLs for P, K, Mg, Ca, Fe, Zn, Mn, and Cu contents in brown rice using introgression lines (ILs) from an interspecific cross of cultivar “Teqing” and Yunnan wild rice (*O. rufipogon*). Using both RILs and backcross introgression lines (BILs) from an inter-subspecific cross Lemont/Teqing, Zhang et al. [16] reported 134 QTLs for the contents of 16 minerals including P, K, Mg, and Ca in brown rice, which were scattered over 39 genomic regions in 12 rice chromosomes. Descalsota-Empleo et al. [11] mapped 50 QTLs for 13 mineral contents in milled rice, including P, K, Na, Mg, and Ca, using two sets of doubled haploid (DH) lines from two inter-subspecific crosses IR64/IR69428 and BR29/IR75862. In addition, Du et al. [14] identified 23 and 9 QTLs for seven mineral contents including P, K, Mg, and Ca in brown rice in the Lingshui and Hangzhou ecological environments, respectively. Of them, only two QTLs for the Mg content were simultaneously identified across two environments, implying that the environment impacts the detection of QTL for grain mineral contents.

Wild species have a high diversity of desirable genes for agronomic traits, and their allelic variation is an important gene pool for genetic improvement [17]. It has been proved that wild rice has much higher contents of minerals than cultivated rice [18, 19], which makes wild rice valuable for biofortification of rice cultivars with enhanced mineral contents.

Dongxiang wild rice (*O. rufipogon* Griff., DWR) is a common wild rice in the northernmost habitat in China and even the world (latitude: 28^o^14' N), which harbors various valuable genes for improving many traits in rice, i.e., biotic and abiotic stresses [20]. A set of backcross inbred lines (BILs) at BC_1_F_5_ derived from an interspecific cross cultivar of Xieqingzao B and an accession of DWR were employed to identify QTLs for planthopper resistance [21], cold tolerance [20], and heavy metal content [22]. In the present study, we conducted QTL mapping for the contents of major macronutrients in brown and milled rice using the BILs and then validated the primarily identified QTLs for Mg and Ca contents using the BILs with increasing genetic homogeneity in the maternal XB background. The main objective was to identify candidate QTLs for macronutrient contents in rice for further fine mapping and cloning. The identified QTLs and favorable alleles from *O. rufipogon* could be used for developing rice varieties with enriched mineral nutrients.

## 2. Materials and Methods

### 2.1. Plant Materials

Two mapping populations derived from an interspecific cross between a cultivar of Xieqingzao B (XB) (*Oryza sativa* L.) and an accession of Dongxiang wild rice (*O*. *rufipogon* Griff.) were used in the present study. The first population, consisting of 202 BC_1_F_5_ BILs previously reported by Chen et al. [23], was used to the initial map QTL for the P, K, Na, Mg, and Ca contents in the present study. XB is an *indica* maintainer line of dwarf-abortive cytoplasmic male sterile line Xieqingzao A, bred by the Rice Research Institute, Anhui Academy of Agricultural Sciences, in the 1980s.

**Figure 1:**
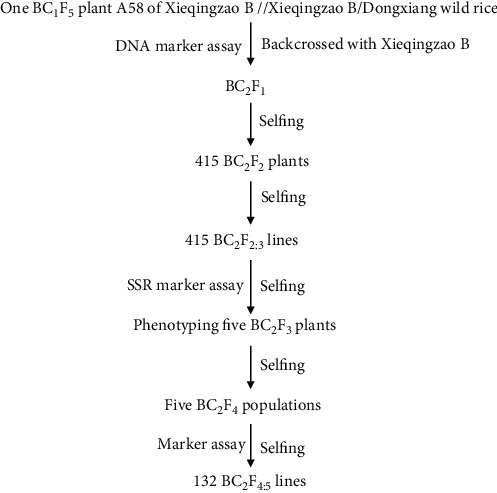
Development of the BC_2_F_4:5_ population.

The second population, consisting of 132 BC_2_F_4:5_ BILs, was used to verify QTL for Mg and Ca contents detected in the BC_1_F_5_ population and identify new QTL in a more homogenous background. This population was developed from a plant in the BC_1_F_5_ population as described and illustrated in Figure 1. The BC_1_F_5_ BIL plant A58 was backcrossed with recurrent parent XB and selfed to generate 415 BC_2_F_2_ plants.

After one generation of selfing, the BC_2_F_2:3_ lines were assayed with 186 polymorphic simple sequence repeat (SSR) markers distributed across 12 chromosomes. The 186 polymorphic SSR markers included 108 SSRs in the original XB/XB//DWR map [23, 24], 78 other SSRs were selected from the Gramene database (http://www.gramene.org/). Of them, 52 SSRs were heterozygous at these loci in the population. Hereafter, these 50 SSRs were used to genotype 415 BC_2_F_2:3_ lines. Based on genotypic and phenotypic data, five BC_2_F_3_ plants from four BC_2_F_2:3_ family lines were selected to generate the BC_2_F_4_ population. Further selection with these markers and agronomic traits resulted in 132 plants from the five BC_2_F_4_ populations (72 plants for each population), and they were selfed to generate 132 BC_2_F_4:5_ lines. The resulting BC_2_F_4:5_ lines were genetically more homogenous in the XB background than their parental BC_1_F_5_ and, thus, were used to verify the QTLs detected in the BC_1_F_5_ population and further identify new QTLs.

### 2.2. Field Experiments

Rice populations were grown in the experimental fields of the China National Rice Research Institute located in both Lingshui, Hainan province (latitude: 18° 30′ N, longitude: 110° 02′ E), and Hangzhou, Zhejiang province (latitude: 30° 04′ N, longitude: 119° 55′ E). The 202 BC_1_F_5_ lines and the recurrent parent XB were grown in Lingshui, Hainan province, in the 2011 winter season (November 2011 to April 2012) (designated as LS11) and Hangzhou, Zhejiang province, in the 2012 summer season (May to October) (designated as HZ12), respectively. The physicochemical properties of the paddy soil in the LS11 and HZ12 trails were listed in Supplementary Table 1. The 132 BC_2_F_4:5_ lines and the parent XB were grown in Hangzhou, Zhejiang province, in the 2016 summer season (May to October) (designated as HZ16) and 2017 summer season (May to October) (designated as HZ17), respectively. In all the field trials, each BIL line consisted of 24 plants in 2 rows, 12 plants per row by transplanting at a spacing specification of 16.7 cm × 26.7 cm between plants. There were no replicates in the BC_1_F_5_ population. For the BC_2_F_4:5_ population, the experiments were conducted under a randomized complete block design with two replicates. Field management and weed control followed the regular agricultural practices. Rice grains of each BIL line were obtained by bulk harvesting the center 10 plants at maturity and threshing manually.

### 2.3. Sample Preparation

Sample grains were stored and air dried at room temperature for three months. For the LS11 and HZ16 trials, 20 g of the grains from each line were dehulled on a Satake sheller (Satake Corporation, Hiroshima, Japan), ground into flour using a Cyclotec 1093 sample grinder (FOSS Tecator, Hoganas, Sweden), and screened through a 0.18 mm mesh sieve. For the HZ12 and HZ17 trials, 30 g of the grains from each line were dehulled and divided in half. One-half of the dehulled rice samples were ground into flour using a Cyclotec 1093 sample grinder and screened through a 0.18 mm mesh; the remaining half were milled for one minute firstly in a Kett Pearlest polisher (Kett Electric Laboratory, Tokyo, Japan) and then grounded using a Cyclotec 1093 sample grinder and screened through a 0.18 mm mesh sieve.

### 2.4. Measurement of the P, K, Na, Mg, and Ca Contents

Approximately 0.5 g flour of each sample was added into a 50 mL polypropylene tube and digested with 8.0 mL 68–70% nitric acid (HNO_3_) and 2.0 mL 70% perchloric acid (HClO_3_) using a digital block digestion system (model ED54, Labtech Inc., Beijing, China). The digestion procedure was as follows: the digestion tube was heated at 80°C for 0.5 hr, 150°C for 2 hrs, and 180°C until 0.5 mL solution remained. After cooling at room temperature, the final digested residues were diluted to 25 mL with double-deionized water. The contents of P, K, Na, Mg, and Ca in the digested solutions were simultaneously determined using an inductively coupled plasma atomic emission spectrometer (IRIS Intrepid II XSP, Thermo Electron Corporation, Milford, MA, USA) according to the manufacturer's instructions. The certified standards (National Institute Center of Standards in China) in flour samples (GBW10010, CRM Rice) were employed to calibrate the contents of five macronutrients. All the measurements were performed in duplication and the average value over two duplications was used for data analysis.

### 2.5. DNA Marker Analysis

Total genomic DNA was extracted following the protocol described by Chen et al. [23]. PCR amplification was performed according to Chen et al. [25]. The PCR products were visualized on 2.5% agarose gel stained with GelRed (Biotium, Hayward, CA, USA) or 6% nondenaturing polyacrylamide gel using silver staining.

### 2.6. Map Construction and Data Analysis

Linkage map of the BC_1_F_5_ population was constructed using simple sequence repeat (SSR) and restriction fragment length polymorphism (RFLP) markers previously by Chen et al. [23] and updated by Huang et al. [24]. The updated map consisted of 149 DNA markers including 108 SSR markers and 41 RFLPs and spanned 1306.4 cM. The linkage map for the BC_2_F_4:5_ population consisted of 186 SSRs which were constructed using MapMaker/Exp 3.0 [26]. The map distance between genetic markers was determined using the Kosambi function and presented in centimorgan.

Statistics were performed for mean, standard deviation (SD), coefficient of variation (CV), skewness, and kurtosis of the phenotypic traits in all trials using the Command DSum of the software Windows QTL Cartographer 2.5 [27]. Pearson's correlation coefficients between the traits were calculated using IBM SPSS Statistics 19. QTL analysis was conducted using the composite interval mapping (CIM) approach of the Windows QTL Cartographer 2.5 [27]. The CIM were performed using the default parameters with backward and forward regressions with a probability threshold of 0.01. The LOD threshold larger than 2.5 was used to claim a putative QTL. The QTLs detected in this study were designated according to the nomenclature reported by McCouch and CGSNL [28].

## 3. Results

### 3.1. Performance of K, P, Na, Mg, and Ca Contents in Brown and Milled Rice

The contents of the five macroelements in brown and milled rice collected from two mapping populations in each trial were presented in Table 1. In all the trials, the contents of the five macronutrients were continuously distributed with the absolute value of skewness and kurtosis less than 1, except for P, K, Na, and Mg contents in milled rice for the HZ12, a typical pattern of quantitative in heritance (Table 1).

For brown rice measured in the BC_1_F_5_ population in both LS11 and HZ12 trials, the average contents of P, K, and Ca in brown rice were higher in the HZ12 trial than in the LS11 trial, but similar for Na and Mg. For brown rice from the BC_2_F_4:5_ population in both HZ16 and HZ17 trials, the average contents of Mg and Ca were higher in the HZ16 trial than in the HZ17 trial (Table 1). These results indicated that the contents of macronutrient in rice were affected by the growing environments, especially the soil (Supplementary Table 1), but the magnitude of influence varied among elements. Pearson correlation analysis indicated that a significantly negative correlation (*r* = −0.45, *P* < 0.01) was only observed for Na between the LS11 and HZ12 trials and no significant correlation was observed for the remaining four macronutrients.

For both the brown and milled rice in the HZ12 and HZ17 trials, the contents of macronutrients in milled rice were lower than those in brown rice. In the HZ12 trial, the average contents of five macronutrients of the BC_1_F_5_ population in milled rice showed a decrease of 57.14% for P, 57.82% for K, 58.14% for Na, 65.28% for Mg, and 57.10% for Ca, when compared with those in brown rice. Similarly, the reductions of 64.15% for P, 63.50% for K, 8.74% for Na, 75.54% for Mg, and 31.24% for Ca, were observed in the milled rice from the brown rice for parental XB. Pearson correlation analysis indicated that a significantly positive correlation was observed for Na, P, and Ca between brown and milled rice, with the correlation coefficients of 0.65 (*P* < 0.01), 0.17 (*P* < 0.05), and 0.16 (*P* < 0.05), respectively, and no significant relationship was observed for the remaining two macronutrients. In the HZ17 trial, the average contents of Mg and Ca in milled rice of the BC_2_F_4:5_ population decreased by 88.12% and 51.99%, respectively, compared to those in brown rice. Likewise for XB, the decreases in the milled rice from the brown rice were 84.14% and 49.29%, respectively, for Mg and Ca contents. The results showed that these macroelements were largely contained in rice bran rather than endosperm. In other words, most of the macroelements in rice are removed during the milling process, which is previously reported [8, 9]. The average contents of Mg and Ca in brown and milled rice of the BC_2_F_4:5_ population in both HZ16 and HZ17 trials were lower than those of the BC_1_F_5_ population in the HZ12 trial. Furthermore, the differences between the average contents of Mg and Ca in brown and milled rice of the BC_2_F_4:5_ population and XB were smaller than those of the BC_1_F_5_ population and XB. This explained that the BC_2_F_4:5_ population was more homogenous in the XB background than the BC_1_F_5_ population.

Correlations between each pair of the macronutrients within a rice type and a trial were presented in Table 2. One significantly negative correlation was observed for Na-Ca in the LS11 trial, while six significantly positive correlations were found in the LS11 trial ranging from 0.75 to 0.98. For the HZ12 trial, there were six significantly positive correlations ranging from 0.34 to 0.97 in either the brown or milled rice, with an odd for Na-Ca, negative (−0.13) in the brown rice but positive (0.41) in the milled rice. All other correlations were either insignificant or low. Overall, the correlations of P-K, P-Mg, and K-Mg were high for all the types of rice samples and trials.

### 3.2. QTLs Detected in the BC_1_F_5_ Population

**Figure 2:**
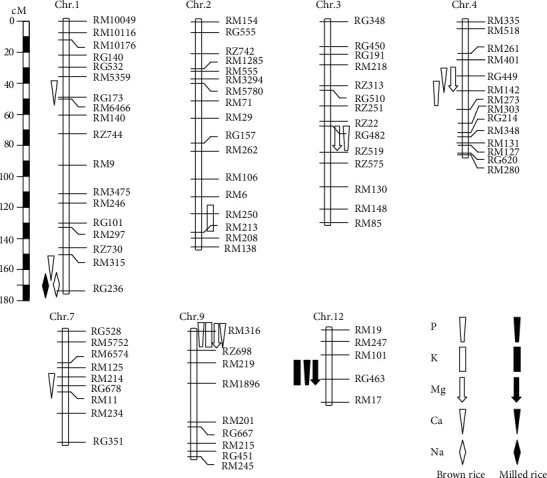
Chromosomal positions of the QTLs conferring P, K, Na, Mg, and Ca contents in brown and milled rice in the BC_1_F_5_ XB^2^/Dongxiang wild rice. QTLs on the right and left hand sides of the chromosomes indicate that they were detected in the LS11 and HZ12 trails, respectively.

In total, 20 QTLs for the five macronutrient contents were claimed from both the LS11 and HZ12 trials in both the brown and rice samples, eight in brown rice showing in the LS11 trial only, seven in brown rice showing in the HZ12 trials only, and five in the milled rice showing in the HZ12 trials (Table 3). The identified QTLs were distributed on eight chromosomes of 1–4, 6–7, 9, and 12 (Figure 2). The proportion of phenotypic variance explained by a single QTL (*R*^2^) ranged from 5.70 to 56.80%. The *qNa1* for Na was found on chromosome 1 flanked by RM315 and RG236 in both brown and milled rice from the HZ12 trial, which was considered as a duplicate QTL. Therefore, the total number of the identified QTLs decreased to 17. Fourteen of these QTLs formed five clusters (Figure 2).

The largest cluster on chromosome 9 consisted of four QTLs, followed by two clusters of three QTLs on chromosomes 4 and 12. In the RM316–RZ698 region on chromosome 9, the XB allele increased P, K, Mg, and Ca contents in brown rice by 276.52 mg/kg, 148.38 mg/kg, 127.30 mg/kg, and 9.38 mg/kg, respectively (Table 3). In the QTL regions in the vicinity of RM142 on chromosome 4, the DWR allele increased P, Mg, and Ca contents in brown rice by 276.47 mg/kg, 101.80 mg/kg, and 22.94 mg/kg, respectively. In the RM101–RG463 region on chromosome 12, the DWR allele increased P, K, and Mg contents in milled rice by 546.40 mg/kg, 312.64 mg/kg, and 199.10 mg/kg, respectively. All three QTLs, *qP12*, *qK12*, and *qMg12*, had their corresponding highest *R*^2^ value of 53.17%, 33.07%, and 56.80%, respectively.

The other two clusters on chromosomes 1 and 3 each included two QTLs. In the RM315–RG236 region on chromosome 1, the DWR allele increased Ca and Na contents in brown rice and Na content in milled rice by 12.84 mg/kg, 3.35 mg/kg, and 3.48 mg/kg, respectively. It is noted that only one QTL exhibited significant effects on the same macronutrient content in both brown and milled rice. The RG482–RZ519 region on chromosome 3 showed significant effects on P and Mg contents in brown rice, with the DWR allele increasing the two traits by 401.37 mg/kg and 172.90 mg/kg, respectively.

In the remaining three regions, one QTL for the K content and two QTLs for the Ca content in each region were detected in the LS11 and HZ12 trials, respectively. They were *qCa1.1* located in the RM5359–RG173 region on chromosome 1, *qK2* in RM250–RM213 on chromosome 2, and *qCa7* in RM214–RG678 on chromosome 7.

### 3.3. Validation of Two QTL Regions in the BC_2_F_4:5_ Population

**Figure 3:**
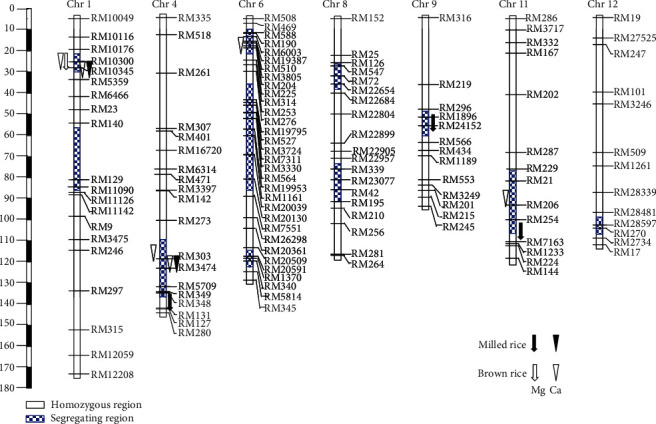
Chromosomal positions of the QTLs conferring Mg and Ca contents in brown and milled rice in the BC_2_F_4:5_ population of Xieqingzao B/Dongxiang wild rice. QTLs on the right and left hand sides of the chromosomes indicate their origination from the HZ16 and HZ17 trials, respectively.

Of 11 segregating regions in the BC_2_F_4:5_ population, QTLs were distributed across 5 regions on chromosomes 1, 4, 6, 9, and 11 (Figure 3; Table 4). Four QTLs for the Mg content and six for the Ca content were identified in both HZ16 and HZ17 trials, including *qCa1.1* and *qCa4.1*, and the others in only one of the trials (Figure 3 and Table 4). The *qCa1.1* for both brown and milled rice Ca contents on chromosome 1 was discovered in the HZ17 trial, making the total number of the identified QTLs down to 9. The *R*^2^ value for brown rice ranged from 13.50% to 17.20% and 19.60% to 19.90% in the HZ16 and HZ17 trials, respectively, and the *R*^2^ value for the milled rice in the HZ17 trial was 17.70% to 63.39%.

Four and three QTLs for the Ca content in both the HZ16 and HZ17 trials (Figure 3 and Table 4) explained 13.5% to 17.2% and 19.60% to 53.38% of the phenotypic variation, respectively. Of them, two segregating QTL regions of the BC_2_F_4:5_ population (Figure 3) were covered or neighbored by the QTLs for the Ca content in the BC_1_F_5_ population, including *qCa1.1* in the interval RM5359–RG173 on chromosome 1 and *qCa4.1* in the interval RG499–RM142 on chromosome 4. The DWR alleles of the *qCa1.1* consistently decreased the brown rice Ca content, while the alleles in *qCa4.1* increased the brown rice Ca content in both the HZ16 and HZ17 trials (Tables 3 and 4). Additionally, the *qCa1.1* showed significant effects on the Ca content in milled rice in the HZ17 trial. *qCa1.1* and *qCa4.1* were consistent across both HZ16 and HZ17 trials, respectively.

The other three QTLs identified in the BC_2_F_4:5_ population were not detected in the BC_1_F_5_ population. One of them, *qCa6.1* in the region of RM588–RM204 on chromosome 6, showed significant effects on the brown rice Ca content in the HZ16 trial, and its DWR allele increased the Ca content by 10.11 mg/kg (Table 4). In the neighboring region of *qCa6.1* between RM276 and RG64 in the BC_1_F_5_ population, *qCa6* for the milled rice Ca content was identified, in which favorable alleles were derived from DWR. At the *qCa4.2* and *qCa11*, dominant XB alleles increased the milled and brown rice Ca contents by 2.98 mg/kg and 5.17 mg/kg, respectively, with *R*^2^ of 53.38% and 13.50%.

One and three QTLs were detected for both the brown and milled rice Mg contents in the HZ16 and HZ17 trials, respectively, with *R*^2^ from 15.50% to 73.39%. The QTL *qMg1* had the XB allele with a partial dominant effect that increased the Mg content in brown rice by 41.76 mg/kg. The remaining three QTLs, *qMg4.1*, *qMg9.1*, and *qMg11* had the enhancing alleles from DWR and showed the overdominant, partially dominant, and additive effects, respectively.

## 4. Discussion

In general, the mineral nutrient content is very low in rice, even though it serves as a major staple food for half the world's population. Therefore, a rice-based diet is associated with mineral deficiency in rice-consuming people. A slight increment of the mineral content in rice should effectively alleviate erupting dietary deficiency worldwide [29]. The increment relies on breeding rice varieties with enhanced mineral nutrient contents using MAS [30]. Identification of QTLs controlling mineral contents in rice is the prerequisite for pyramiding of QTL for mineral biofortification with MAS.

### 4.1. Genetic and Environmental Effects on Macronutrient Content QTL Detection

Using various mapping populations from the same cross phenotype across environments could help eliminate genetic and environmental noise for dissecting consistent QTL. In the present study, we used a same interspecific cross between Xieqingzao B (*Oryza sativa* L.) and Dongxiang wild rice (*O*. *rufipogon* Griff.) to generate two populations of BC_1_F_5_ and BC_2_F_4:5_ BILs for QTL analysis across two environments and two seasons, respectively. As a result, 17 QTLs for macronutrient contents in brown and milled rice were initially identified in BC_1_F_5_ BILs, and then, 9 QTLs for Ca and Mg contents were in BC_2_F_4:5_ BILs in which the background should be more homogeneous than that of the BC_1_F_5_ BILs. In addition, more QTLs with higher statistical significance and effectiveness were resulted from the BC_2_F_4:5_ BILs than from the BC_1_F_5_ BILs. The higher significance and effectiveness might be due to a high genetic homogeneity of 72.04% at 186 marker loci in the XB genetic background, indicating that BC_2_F_4:5_ BILs are more appropriate to detect QTL with smaller effect than BC_1_F_5_ BILs. The result is in accord with the findings reported by Zhang et al. [16].

Mineral contents in rice were influenced by multiple factors [14, 22], especially the soil. The climate in Lingshui, Hainan, is a typical tropical monsoonal, with sufficient sunshine and abundant rainfall, while that in Hangzhou, Zhejiang, is a humid subtropical. The physicochemical properties of the soil (Supplementary Table 1) were obviously different at two distinct ecological locations. Accordingly, the contents of macronutrients in brown rice were different between LS11 and HZ12 trials. Taking Ca content in brown rice as an example, the values in the HZ12 trial ranged from 114.4 to 313.4 mg/kg^−1^ with an average content of 197.4 mg/kg^−1^, which were higher than the values ranging from 61.7 to 208.6 mg/kg^−1^ with a mean value of 133.0 mg/kg^−1^ in the LS11 trial. Consistent with previous studies [14, 22, 31], 17 QTLs for macronutrient contents in BC_1_F_5_ BILs were all environment specific. These results suggested that different environmental factors, especially the soil, affected the accumulations of the macronutrients as well as QTL detection. However, it is notable that two QTLs, *qCa1.1* and *qCa4.1*, were validated with constant allelic directions by identification across populations and seasons. These environment-specific and/or stable QTLs across environments with consistent allelic effects are of great importance, which provided good candidates for rice biofortification breeding and further gene cloning.

### 4.2. QTL Cluster for Macronutrient Contents

QTL clusters for multi-macronutrient contents in this study are in line with previous reports [5, 11, 14–16]. In total, 14 and 4 of the QTLs in the BC_1_F_5_ and BC_2_F_4:5_ populations were clustered over five and two genomic regions distributed on six chromosomes, respectively. Among them, six regions were also reported in previous studies and the remaining regions were novel. Three genomic regions of eight QTL clusters were especially important. One super QTL cluster on chromosome 9 contained *qP9*, *qK9*, *qCa9*, and *qMg9*, of which *qK9* was also identified for the K content in brown rice reported previously [16]. The second QTL cluster *qP12*/*qK12*/*qMg12* was within the interval RM101–RG463 on chromosome 12. Three QTLs in this region, *qP12*, *qK12* and *qMg12*, all had the largest *R*^2^ and favorable alleles from *O*. *rufipogon*, of which *qP12* and *qMg12* were reported in previous studies [15, 16, 32]. The remaining QTL cluster *qCa1.1*/*qMg1.1* was adjacent to the interval in the vicinity of RM5359 on chromosome 1, of which *qCa1.1* was across two mapping populations and three trials, providing a good candidate for enhancing the Ca content in both the brown and milled rice. In addition, further fine mapping and cloning are required to confirm the allelic relationships of these QTL clusters.

The significant correlations of macronutrient contents with each other were observed, and those correlations were the same in both the brown and milled rice samples. Notably, the highly positive correlations were found for each pair of K, P, Mg, and Ca contents in the brown and milled rice samples in both HZ12 and LS11 trials. Their high correlations have been previously reported [5, 16]. Accordingly, two QTL clusters for K-P-Mg contents in brown and milled rice were colocated on chromosomes 9 and 12, respectively. These QTL clusters demonstrate that the pleiotropic gene or tightly linked genes controlling different macronutrient contents were located on the same chromosomal region, probably because these different macronutrients are involved in the common regulatory pathways. Once the synergistic associations for these macronutrients are confirmed, the improvement of the macronutrient contents could be very effective because a single effort in rice breeding practices could result in an enhancement of the multi-macronutrient content simultaneously.

### 4.3. Comparison of QTLs for Macronutrient Contents between Present and Previous Studies

Based on the physical position of flanking markers, we compared the similarity for the QTLs identified in the present study with earlier reports for the same macronutrient within a physical distance of 1 Mb. Of 24 QTLs, 14 were coincident with previous studies and 10 were novel QTLs. *qP12* and *qK9* accorded with a QTL related to the P content [15, 16, 32] and K content [16], respectively, in previous reports. The *qMg1* coincided with *qMg1.1* for the Mg content in milled rice [11], *qMg3* with *qMg3-1* [15] and *qMg3.2* [5], *qMg9* with *qMg9.1* for brown rice [5] and *qMg9* for milled rice [33], *qMg11* with *qMg11.1* [11] and *qMg11* [33], and *qMg12* with *qMg12-1* [15]. *qCa1.1* and *qCa1.2* accorded with *qCa1.1* for the Ca content in milled rice [34] and a QTL for brown rice [35, 36], respectively, *qCa4.2* with a QTL for the Ca content in brown rice [15, 16], *qCa6.1* with *qCa6.2* and *qCa6* for brown rice [5, 37] and *qCa6* for milled rice [13], *qCa9* with a QTL for the Ca content in brown rice in earlier studies [15, 36], *qCa11* with a QTL for brown rice reported by Du et al. [14], and *qNa1* with *qNa1* for the Na content [37]. These QTLs across diverse genetic backgrounds and environments are considered as consensus QTLs. They are worthy of exploiting the full potential for biofortifying rice with enriched macronutrient in breeding practices, while effort should be focused on the identification of molecular markers tightly linked to the target QTLs for MAS breeding. Moreover, these consensus QTLs in various studies are also good candidates for verification of allelic effects, fine mapping, and cloning.

A large number of genes involved in mineral absorption and translocation have been isolated and characterized in rice. As expected, some potential genes related to macronutrient uptake, transportation, and homeostasis in rice were within the chromosomal regions of the QTLs in present study. These QTL regions harbor the following potential genes, such as *OsPAP10c* for P, *OsHAK2* for Na, *OsCNGC4*, *OsCNGC5*, *OsKCO3*, and *OsCNGC17* for K; *OsMGT3* for Mg; and *OsEFCAX1*, *OsCam1-3*, *OsCAX3*, *OsCCX5*, and *OsCML6* for Ca. Interestingly, *OsEFCAX1* (LOC_Os01g11414) and *OsCam1-3* (LOC_Os01g16240) for Ca are present within the *qCa1.1/qMg1* regions consistently across multiexperiments of the present study (Figures 2 and 3), which are involved in calcium-mediated signaling and homeostasis and expressed in all tissues including seeds [38, 39]. It would be important to generate near-isogenic lines differing in alleles at these gene loci on the XB genetic background for investigation of the relationships with the QTLs identified in the present study.

### 4.4. Utilization of Dongxiang Wild Rice for Rice Biofortification Breeding

To satisfy the growing demand of food and dietary nutrition security in the rice-consuming population, more and more attention has been focused on enhancing the contents of essential nutrients while decreasing the concentrations of toxic metals in rice grain. However, the complex interactions between toxic elements and essential nutrients add difficulty to this global effort [40]. Previously, we have used the same BC_1_F_5_ BILs to map QTL for grain yield with contributing components [24]. In the previous and present studies, we have reported eight QTLs for yield components and six QTLs for macronutrient contents which distribute over four chromosomal regions or clusters on chromosomes 4, 6, 7, and 12. Two major clusters on chromosomes 4 and 12 holding four QTLs are *qTGW-12* and *qMg12*/*qP12*/*qK12* and *qSF-4*/*qNFGP-4*/*qGYD-4* and *qCa4.2*. These two clusters demonstrate that the DWR alleles have synergistic effects for yield and macronutrient traits in the QTL regions on chromosomes 4 and 12. Many other reports have revealed the synergistic effects, as well [31, 34, 41]. The remaining two clusters on chromosomes 6 and 7 harbor three QTLs, *qNFGP-6*/*qSF-6*, *qCa6.1*, and *qNFSP-7*/*qTFGP-7*/*qCa7*. However, the DWR alleles in the clustered QTLs on chromosomes 6 and 7 showed the opposite allele effects on the macronutrient content and yield components. These results demonstrate that the *O*. *rufipogon* alleles in favor of some traits may be unfavorable to the others. These deleterious associations between grain mineral nutrients and yield traits have been reported previously [5, 11, 16, 42]. Interruption of deleterious linkages should be done before those desirable genes could be transferred from *O. rufipogon* into *O. sativa* in cultivar improvement.

Up to now, much efforts have been made to exploit favorable alleles from wild rice species for grain quality enhancement [43], grain yield, and resistance improvement [44]. Wild rice is rich in some essential nutrients in comparison of cultivated rice [18, 19]. Dongxiang wild rice is the progenitor of cultivated rice, having numerous favorable alleles [21, 45]. These alleles are widely utilized in genomic and breeding studies for improvement of important agronomic traits. Notably, 11 of 17 (64.70%) and 5 of 9 (55.55%) QTLs in the BC_1_F_5_ and BC_2_F_4:5_ BILs have favorable alleles from *O. rufipogon* for enrichment of macronutrient contents. Similarly, previous studies showed that three AA-genome wild rice species, *O. rufipogon*, *O. nivara*, and *O. meridionalis*, have favorable alleles of 83.87% (26 of 31 QTLs), 83.33% (25 of 30 QTLs), and 100% (4 of 4 QTLs), respectively, for gain mineral nutrient contents [15, 46, 47]. These results suggest that wild relatives of rice cultivar may greatly contribute to improving grain mineral nutrient contents in cultivated species of *O*. *sativa*. They have precious potentials for the biofortification of cultivated rice with enhanced grain mineral contents. Introgression of favorable alleles from *O*. *rufipogon* may introduce some unfavorable allele into elite rice cultivars because of their tight linkage. It becomes important to interrupt these types of linkage for breeding rice varieties for integrating grain yield with mineral traits.

## 5. Conclusions

A total of 24 QTLs for the contents of five macronutrients in rice were detected using two BIL populations derived from the same interspecific cross. Dongxiang wild rice contributed 65.38% (16 out of 24) favorable QTL alleles for the improvement of the grain macronutrient content in future breeding. Two QTLs *qCa1.1* and *qCa4.1* with consistent allelic directions across different populations and/or trials should be taken into account in grain nutrient biofortification through QTL pyramiding.

## Figures and Tables

**Table 1:** Phenotypic performance of macronutrient contents in brown and milled rice in two sets of BIL populations derived from interspecific cross of Xieqingzao B/Dongxiang wild rice.

Population	Element	Trial^∗^	Tissue	Mean (mg/kg)	SD	CV	Range	Skewness	Kurtosis	XB (mg/kg)
BC_1_F_5_	P	LS11	Brown rice	3785.6	803.6	0.14	2096.0–5868.0	0.34	−0.39	3550.0
		HZ12	Brown rice	3956.8	642.9	0.11	2112.0–5868.0	0.49	0.36	2848.0
			Milled rice	1695.8	439.0	0.13	988.0–3426.0	1.59	2.63	1021.0
	K	LS11	Brown rice	2109.9	476.6	0.14	1089.0–3384.0	0.25	−0.37	2102.0
		HZ12	Brown rice	2475.9	388.9	0.11	1507.0–3568.0	0.20	−0.005	1810.0
			Milled rice	1044.3	286.6	0.13	522.0–2214.0	1.28	2.29	661.0
	Mg	LS11	Brown rice	1442.4	361.3	0.15	696–2416.0	0.39	−0.29	1461.0
		HZ12	Brown rice	1339.4	251.9	0.11	732.0–2218.0	0.67	1.01	1068.0
			Milled rice	465.0	163.1	0.15	214.0–1062.0	1.45	1.94	261.0
	Ca	LS11	Brown rice	133.0	29.1	0.14	61.7–208.6	0.29	−0.42	121.9
		HZ12	Brown rice	197.4	42.9	0.14	114.4–313.4	0.37	−0.36	179.1
			Milled rice	84.7	33.7	0.17	3.3–201.8	0.18	0.31	123.2
	Na	LS11	Brown rice	18.75	9.23	0.22	3.45–41.72	0.21	−1.14	22.12
		HZ12	Brown rice	14.36	6.41	0.18	0.08–36.22	0.54	0.10	10.30
			Milled rice	6.01	4.44	0.17	0.06–25.96	1.10	1.64	9.40

BC_2_F_4:5_	Mg	HZ16	Brown rice	1383.9	90.6	0.07	1207.3–1638.5	0.61	0.17	1257.0
	Ca		Brown rice	148.2	18.3	0.12	115.2–201.4	0.54	−0.35	134.6
	Mg	HZ17	Brown rice	1339.5	31.5	0.02	1275.5–1397.6	−0.18	−0.81	1332.3
			Milled rice	159.1	46.6	0.29	89.7–246.7	0.31	−1.36	211.2
	Ca	HZ17	Brown rice	137.7	12.1	0.09	117.4–159.7	0.05	−1.13	141.8
			Milled rice	66.1	7.4	0.11	54.0–80.7	0.26	−0.95	71.9

^∗^LS11: 2011 winter season in Lingshui; HZ12: 2012 summer season in Hangzhou; HZ16: 2016 summer season in Hangzhou; HZ17: 2017 summer season in Hangzhou.

**Table 2:** Correlation coefficients among five macroelement contents in the LS11 and HZ12 trials.

Trial	Tissue	Element	K	Mg	Ca	Na
LS11	Brown rice	P	0.94^∗∗^	0.98^∗∗^	0.78^∗∗^	−0. 11
		K		0.93^∗∗^	0.76^∗∗^	−0. 12
		Mg			0.75^∗∗^	−0. 12
		Ca				0.31^∗∗^

HZ12	Brown rice	P	0.90^∗∗^	0.95^∗∗^	0.42^∗∗^	0.05
		K		0.86^∗∗^	0.52^∗∗^	−0.08
		Mg			0.36^∗∗^	0.12
		Ca				−0.13
	Milled rice	P	0.91^∗∗^	0.97^∗∗^	0.34^∗∗^	−0.01
		K		0.92^∗∗^	0.43^∗∗^	0.05
		Mg			0.38^∗∗^	0.03
		Ca				0.41^∗∗^

“^∗^” and “^∗∗^” represent significance levels of *P* < 0.05 and 0.01, respectively.

**Table 3:** QTLs for macroelement contents in brown and milled rice detected in the BC_1_F_5_ BIL population.

Trial	Tissue	Element	QTL	Interval	*LOD*	*A* ^∗^	*R* ^2^%	Previous reports
LS11	Brown rice	P	*qP3*	RG482-RZ519	3.59	401.37	11.36	
			*qP9*	RM316-RZ698	3.04	−276.52	8.73	
		K	*qK2*	RM250-RM213	2.53	175.05	7.30	
			*qK9*	RM316-RZ698	2.49	−148.38	7.03	[16]
		Mg	*qMg3*	RG482-RZ519	3.45	172.90	10.30	[5, 15]
			*qMg9*	RM316-RZ698	3.45	−127.30	9.10	[5, 32]
		Ca	*qCa9*	RM316-RZ698	2.60	−9.38	7.47	[15, 35]

HZ12	Brown rice	P	*qP4*	RM142-RM273	2.66	276.47	7.67	
		Mg	*qMg4*	RG449-RM142	2.52	101.80	6.70	
		Ca	*qCa1.1*	RM5359-RG173	3.47	−14.58	6.94	[34]
			*qCa1.2*	RM315-RG236	3.02	12.84	5.98	[35, 36]
			*qCa4.1*	RG449-RM142	5.24	22.94	13.00	
			*qCa7*	RM214-RG678	3.02	−19.89	6.46	
		Na	*qNa1*	RM315-RG236	2.59	3.35	10.59	[37]
	Milled rice	P	*qP12*	RM101-RG463	5.06	546.40	53.17	[15, 16, 32]
		K	*qK12*	RM101-RG463	3.02	312.64	33.07	
		Mg	*qMg12*	RM101-RG463	4.48	199.10	56.80	[15]
		Na	*qNa1*	RM315-RG236	2.87	3.48	22.37	[37]

*A* ^∗^ indicates an additive effect of replacing a Xieqingzao B allele by a Dongxiang wild rice allele; *R*^2^ indicates the proportion of phenotypic variance explained by the QTL effect.

**Table 4:** QTLs for Mg and Ca contents in brown and milled rice detected in the BC_2_F_4:5_ population.

Trial	Tissue	Element	QTL	Interval	LOD	*A*∗	*D*	*R* ^2^	Previous reports
HZ16	Brown rice	Mg	*qMg1*	RM10300-RM10345	4.86	−41.76	−18.81	15.50	[11]
		Ca	*qCa1.1*	RM10300-RM10345	8.73	−9.00	−4.36	15.10	[34]
			*qCa4.1*	RM273-RM303	3.12	17.88	−4.93	13.60	
			*qCa6.1*	RM588-RM204	9.62	10.11	9.66	17.20	[5, 13, 37]
			*qCa11*	RM21-RM206	7.27	−5.17	10.56	13.50	[14]

HZ17	Brown rice	Ca	*qCa1.1*	RM10300-RM10345	2.62	−11.93	7.34	19.90	[34]
			*qCa4.1*	RM273-RM303	2.53	6.61	8.60	19.60	
	Milled rice	Mg	*qMg4.1*	RM348-RM131	3.38	33.59	−81.32	17.90	
			*qMg9.1*	RM1896-RM24152	2.91	65.49	−19.67	17.70	
			*qMg11*	RM254-RM7163	14.56	45.72	−7.32	63.39	[11, 33]
		Ca	*qCa1.1*	RM10300-RM10345	2.54	−6.75	5.68	18.77	[34]
			*qCa4.2*	RM348-RM131	2.88	−2.98	13.58	53.38	[15, 16]

*A* ^∗^ indicates an additive effect of replacing a Xieqingzao B allele by a Dongxiang wild rice allele; *R*^2^ indicates the proportion of phenotypic variance explained by the QTL effect.

## Data Availability

All the relevant data have been incorporated into the original research manuscript.

## Supplementary Materials

Supplementary Materials Supplementary Table 1 The physicochemical characteristics of the soil in the experimental fields in LS11 and HZ12.
