# Supplementary material for: Identification and Validation of QTLs for Macronutrient Contents in Brown and Milled Rice Using Two Backcross Populations between Oryza sativa and O. rufipogon
Source: Biomed Res Int. 2021 Jun 11;2021:5561734. doi: 10.1155/2021/5561734 (PMC8214480; doi:10.1155/2021/5561734)
Supplement: Supplementary Materials — Supplementary Table 1 The physicochemical characteristics of the soil in the experimental fields in LS11 and HZ12. [file 5561734.f1.docx]

**SUPPLEMENTARY Description:**

**Supplementary Table 1. The physicochemical characteristics of the soil in the experimental fields in the LS11 and HZ12.**
